# A Preview of Selected Articles

**DOI:** 10.1002/sctm.20-0437

**Published:** 2020-10-22

**Authors:** Stuart P. Atkinson

**Affiliations:** ^1^ Centro de Investigación Príncipe Felipe Valencia Spain

Injury to organs such as the skin triggers an initially adaptive fibrogenic wound healing response involving the replacement of normal parenchymal tissue with connective tissue; however, the prolonged activation of the fibrotic response can prompt the accumulation of excessive amounts of fibrotic tissue and the formation of scar tissue, which can disrupt normal organ function.^1^ A variety of cells interact during wounding healing/fibrosis, including inflammatory cells and effector cells (e.g., fibroblasts and myofibroblasts), with the transforming growth factor‐beta (TGF‐β) signaling pathway playing a controlling role in almost all cases.^2^ In the skin, the reduced elasticity and dermal thickening associated with fibrosis can cause pain and disfigurement and significantly impair tissue function.^3^ Autologous fat grafting represents one proven means to treat skin fibrosis in breast cancer patients receiving radiotherapy, with adipose‐resident mesenchymal stem cells (MSC) thought to represent the primary drivers of regenerative/reparative response through the secretion of growth factors with adipogenic, angiogenic, and antifibrotic effects. Ongoing research is currently exploring the development of enhanced fat grafting or MSC‐based therapies and other exciting new treatment strategies that can reduce fibrosis and improve wound healing outcomes. In our first Featured Article published this month in *STEM CELLS Translational Medicine*, Borrelli et al. report on the antifibrotic activity of CD74‐expressing adipose‐derived MSCs and their application in fat grafts as an improved treatment for radiation‐induced fibrosis of the skin.^4^ In a Related Article published in *STEM CELLS*, Doeser et al. described how the transient induction of induced pluripotent stem cell (iPSC)‐associated reprogramming factors during wound healing prompted a reduction in both fibrotic activity and scar tissue formation.^5^

The tumorigenic potential of undifferentiated self‐renewing cells represents one of the major problems facing the development of regenerative strategies involving human embryonic stem cells (ESCs), iPSCs, and their differentiated derivatives.^6^ Indirect approaches to improve the safety of said regenerative approaches involve the development of enhanced differentiation protocols or the use of “master banks” of apparently safe human pluripotent stem cells (PSCs); however, these approaches cannot completely eliminate any tumorigenic risk.^7^ While more direct approaches to the elimination of undifferentiated PSCs tend to focus on the pre‐transplant stage, others aim to address the potential tumorigenicity of cells post‐transplantation. These alternative approaches include the engineering of PSCs to contain genetic safety switches or “suicide genes,” a strategy extensively explored in cancer gene therapy, that can selectively and inducibly eliminate pluripotent and tumorigenic cells. Suicide gene technologies include the overexpression of CD20 to promote elimination by complement/antibody‐dependent cellular cytotoxicity, a metabolic suicide gene system employing the herpes simplex virus thymidine kinase gene with the ganciclovir prodrug, and a dimerization‐induced caspase‐9 (iCASP9) suicide gene system.^8^ In our second Featured Article published this month in *STEM CELLS Translational Medicine*, Shi et al. describe the improved design and subsequent evaluation of the iCASP9 suicide strategy, which they hope will improve the safety and efficacy of iPSC‐based regenerative therapies.^9^ In a Related Article published in *STEM CELLS*, Ide et al. reported on a novel method for efficiently generating lentiviral vectors that can specifically target potentially tumorigenic undifferentiated human PSCs and highlighted the utility of an approach based on the herpes simplex virus *thymidine kinase* gene driven by the survivin promoter.^10^

## FEATURED ARTICLES

### Enhanced Adipose‐Derived MSC Therapy for Fibrosis Treatment

Recent research describing a role for cells expressing the CD74 cell surface marker in the inhibition of liver fibrosis and spontaneous lung injury in mouse models^11^, ^12^ suggested to researchers led by Derrick C. Wan (Stanford University School of Medicine, Stanford, CA, USA) that CD74‐expressing adipose‐derived MSCs may possess enhanced antifibrotic and proregenerative capabilities. In their recently published *STEM CELLS Translational Medicine* article,^4^ Borrelli et al. sought to explore this subpopulation of cells as an enhanced means to treat radiation‐induced fibrosis, which can distort skin form, impair skin function, and negatively impact the quality‐of‐life of those affected. Fascinatingly, initial in vitro analysis established that CD74‐sorted MSCs expressed higher levels of crucial growth factor proteins, including hepatocyte growth factor (HGF), fibroblast growth factor 2 (FGF2), and TGF‐β3, and induced less profibrotic extracellular matrix components from stimulated dermal fibroblasts. Moving in vivo to a mouse model of radiation‐induced fibrosis, the team then demonstrated that fat grafts enriched for CD74‐expressing MSCs reversed many of the signs of fibrosis and promoted significant improvements to the skin, including a reduction in stiffness, dermal thickness, and collagen content in the overlying skin, and a decrease in the proportion of more fibrotic dermal fibroblasts. With these fascinating findings, the authors provide considerable evidence that treatment with CD74‐expressing adipose‐derived MSCs may represent an efficient means to address fibrotic remodeling following radiation‐induced fibrosis in human patients.

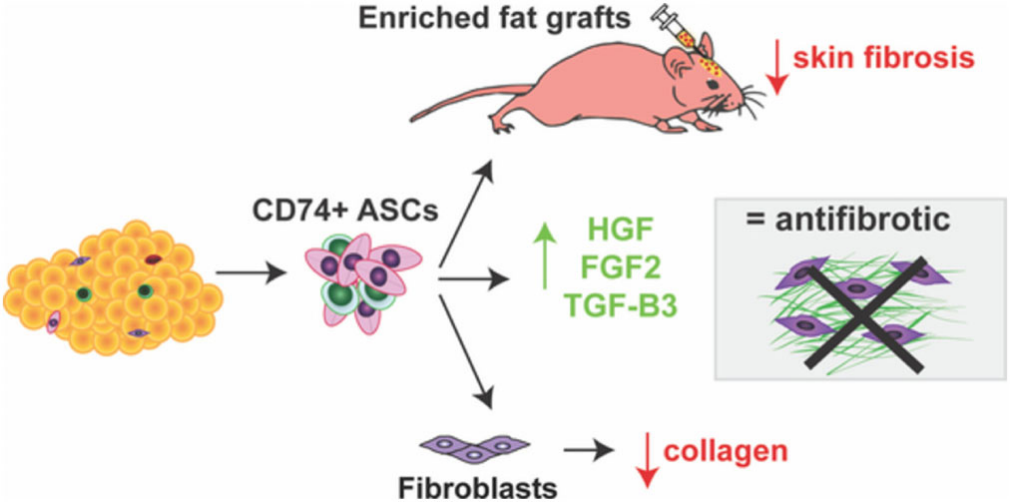

https://doi.org/10.1002/sctm.19-0317

### Inducible Suicide Gene Safety System for iPSC‐Based Regenerative Therapies

The iCASP9 dimerization‐induced suicide gene system has the potential to efficiently remove residual and potentially problematic undifferentiated iPSCs to promote the development of safe and effective regenerative therapies; however, non‐specific genomic integration of the iCASP9 system in human iPSCs through lentiviral infection can induce problems, which include oncogenic alterations and iCASP9 silencing.^13^, ^14^, ^15^ Recently, researchers from the laboratory of Aaron J. Carman (InVitro Cell Research, LLC, New York USA) aimed to improve this highly useful approach by exploring the integration of the iCASP9 system into a human genome safe harbor and evaluating optimal promoter sequences to drive the iCASP9 system. Reporting recently in *STEM CELLS Translational Medicine*,^9^ Shi et al. discovered that the *AAVS1* locus in the *PPP1R12C* gene possessed significant advantages for the TALEN‐mediated installation of the iCASP9 system when compared with the *CCR5* and *hROSA26* loci. While the EF1α promoter becomes silenced and the endogenous promoter of the *PPP1R12C* is too weak, the CAG promoter induces strong and stable iCASP9 expression to rapidly promote the in vitro elimination of iPSCs and their differentiated derivatives (including MSCs and chondrocytes) following small molecule‐mediated activation. Finally, and most excitingly, administering the small‐molecule iCASP9 activator to highly immunodeficient mice bearing teratomas induced by iPSCs carrying the CAG‐controlled iCASP9 system prompted dramatic tumor shrinkage. Overall, the authors describe much‐needed improvements to the existing iCASP9 suicide gene technology in a study that hopes to pave the way toward the development of safe and effective human PSC‐based therapeutics.

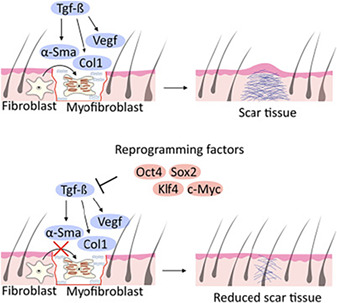

https://doi.org/10.1002/sctm.20-0007

## RELATED ARTICLES

### Transient Expression of Reprogramming Factors Reduces Fibrosis and Scar Formation

As the in vivo expression of the OCT4, SOX2, KLF4, and C‐MYC (OSKM) reprogramming factors improves signs of senescence in progeria mice,^16^ enhances tissue regeneration in aged mice,^16^ and promotes the repair of traumatic brain injury,^17^ many have posited partial reprogramming as a general strategy for the regeneration of damaged tissues. Researchers led by Hans R. Schöler and Guangming Wu (Max Planck Institute for Molecular Biomedicine, Münster, Germany) performed cutaneous wound healing experiments in OSKM‐inducible transgenic mice to evaluate if the transient expression of iPSC‐associated reprogramming factors could influence the degree of fibrosis and scarring. Reporting in *STEM CELLS*,^5^ Doeser et al. discovered that the selective and transient expression of OSKM in mouse cutaneous wounds prompted a reduction in wound contraction, fibroblast migration, fibroblast‐to‐myofibroblast transdifferentiation (a crucial step in the pathogenesis of fibrosis), and the expression of profibrotic genes via the suppression of the TGF‐β signaling pathway. In turn, these advantageous alterations improved wound healing and significantly inhibited scar tissue formation during wound healing without inducing any signs of tumorigenesis; however, the authors failed to observe any impact of transient OSKM expression on the level of senescence‐associated gene expression. In summary, this fascinating study provided further evidence that partial reprogramming by the transient induction of the OSKM factors could represent a safe and effective means to promote the scarless regeneration of damaged tissues and potentially reduce the impact of fibrosis in affected organs.

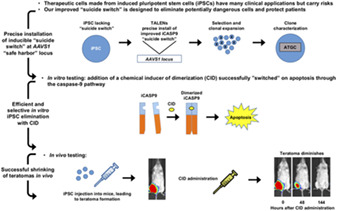

https://doi.org/10.1002/stem.2842

### Targeting the Tumorigenic Progeny of Pluripotent Cells Through the Development of Novel Lentiviral Vectors

In the quest to ensure the safety and efficacy of human PSC‐based regenerative therapies, researchers from the laboratory of Ken‐ichiro Kosai (Kagoshima University Graduate School of Medical and Dental Sciences, Japan) previously developed an adenoviral conditional targeting method for securely isolating target PSCs^18^ and an oncolytic virus‐based strategy to specifically and efficiently kill undifferentiated PSCs.^7^ In the team's subsequent *STEM CELLS* article,^10^ Ide et al. described a novel two‐plasmid system that allowed for the generation and evaluation of diverse tumorigenic cell‐targeting lentiviral vectors expressing suicide genes under an optimized promoter. Employing this approach, the authors described how ganciclovir treatment displayed robust cytotoxicity only in PSCs transduced with a lentiviral vector containing the herpes simplex virus *thymidine kinase* gene downstream of the promoter for the cancer‐ and PSC‐specific survivin gene. Of note, ganciclovir administration promoted the death of almost all undifferentiated PSCs, even though only 5% of the cells expressed the transgenes, thereby suggesting that a potent bystander effect should represent an advantage in clinical situations. Furthermore, the same system also completely inhibited the growth of transduced PSC‐derived teratomas after the administration of ganciclovir to mice with no evidence of any unwanted side‐effects. Overall, the authors hoped that both their novel methodology and the description of an effective suicide gene strategy may pave the way toward safe and effective human PSC‐based regenerative therapies.

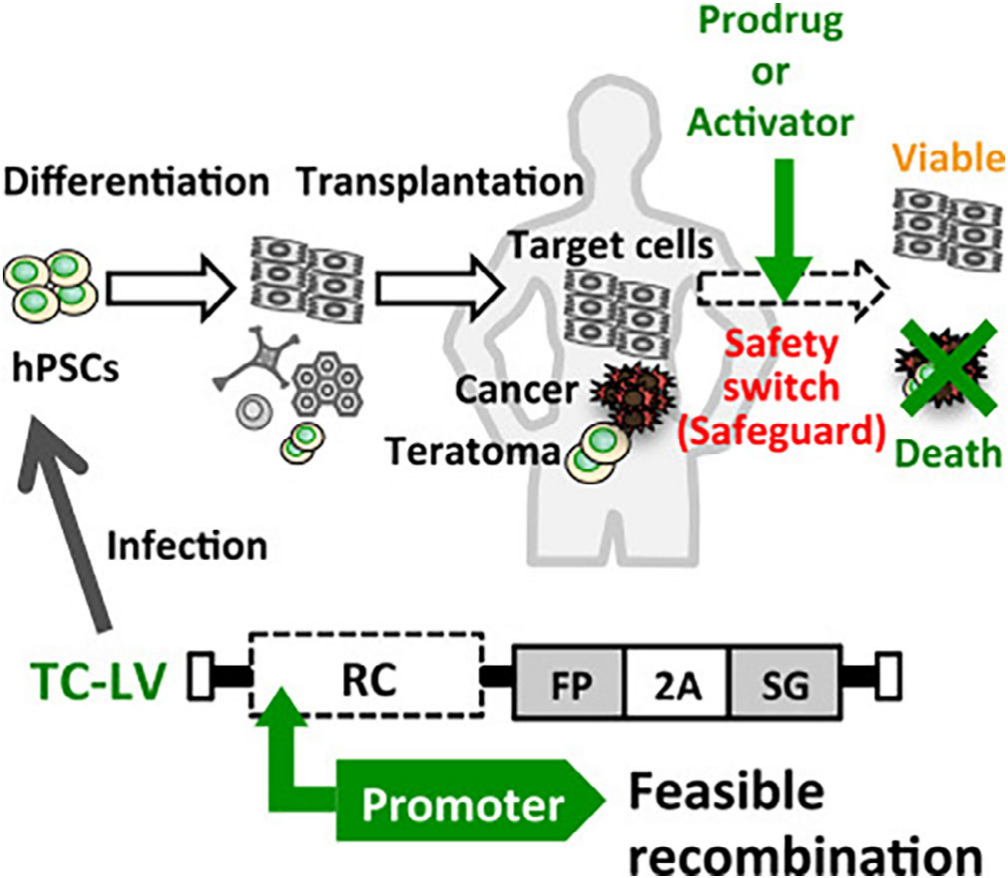

https://doi.org/10.1002/stem.2725
